# Ecological monitoring of emotional intensity, variability, and instability in individuals with schizophrenia spectrum disorders: Results of a multicentre study

**DOI:** 10.1002/mpr.1992

**Published:** 2023-09-20

**Authors:** Cristina Zarbo, Manuel Zamparini, Alessandra Patrono, Cosima Calini, Philip D. Harvey, Letizia Casiraghi, Massimo Clerici, Matteo Malvezzi, Matteo Rocchetti, Fabrizio Starace, Giovanni de Girolamo, DiAPAson Collaborators

**Affiliations:** ^1^ Department of Psychology University of Milano Bicocca Milan Italy; ^2^ Unit of Epidemiological and Evaluation Psychiatry IRCCS Istituto Centro San Giovanni di Dio Fatebenefratelli Brescia Italy; ^3^ Department of Molecular and Translational Medicine University of Brescia Brescia Italy; ^4^ Department of Medicine and Surgery University of Milan Bicocca Monza Italy; ^5^ Department of Psychiatry and Behavioral Sciences University of Miami Miller School of Medicine Miami Florida USA; ^6^ Department of Clinical Sciences and Community Health University of Milan Milan Italy; ^7^ Department of Brain and Behavioural Sciences University of Pavia Pavia Italy; ^8^ Department of Mental Health and Dependence ASST of Pavia Pavia Italy; ^9^ Department of Mental Health and Dependence AUSL of Modena Modena Italy

**Keywords:** emotional instability, emotional variability, experience sampling method, prospective design, psychosis

## Abstract

**Background:**

Evaluating emotional experiences in the life of people with Schizophrenia Spectrum Disorder (SSD) is fundamental for developing interventions aimed at promoting well‐being in specific times and contexts. However, little is known about emotional variability in this population. In DiAPAson project, we evaluated between‐ and within‐person differences in emotional intensity, variability, and instability between people with SSD and healthy controls, and the association with psychiatric severity and levels of functioning.

**Methods:**

102 individuals diagnosed with SSD (57 residential patients, 46 outpatients) and 112 healthy controls were thoroughly evaluated. Daily emotions were prospectively assessed with Experience Sampling Method eight times a day for a week. Statistical analyses included ANOVA, correlations, and generalized linear models.

**Results:**

Participants with SSD, and especially residential patients, had a higher intensity of negative emotions when compared to controls. Moreover, all people with SSD reported a greater between‐person‐variability of both positive and negative emotions and greater intra‐variability of negative emotions than healthy controls. In addition, the emotion variability in people with SSD does not follow a linear or quadratic trend but is more “chaotic” if compared to controls.

**Conclusions:**

Adequate assessments of positive and negative emotional experiences and their time course in people with SSD can assist mental health professionals with well‐being assessment, implementing targeted interventions through the identification of patterns, triggers, and potential predictors of emotional states.

## 1 INTRODUCTION

Schizophrenia Spectrum Disorders (SSD) are a group of mental disorders characterized by heterogeneous positive (i.e., hallucinations, delusions, and disorganization) and negative (anhedonia, avolition, poverty of thought) symptoms, as well as cognitive and motivational dysfunctions (Correll & Schooler, 2020; Kahn et al., 2015; Winship et al., 2019). Emotion processing (i.e., the correct interpretation and coding of emotions through expressions and behavior) has long been known to be highly impaired in this population (Berenbaum & Oltmanns, 1992; Kring et al., 1993; Kring & Neale, 1996). As people with SSD seem to experience generally increased emotional experiences ranging from general distress/negative affect to major depression (Upthegrove et al., 2016), available data supports the notion that these individuals have difficulties in regulating negative emotions and distress. Indeed, they have an increased sensitivity to small daily life stressors (Myin‐Germeys et al., 2001), resulting in a pattern of emotional variation that differs from healthy individuals.

In this context, both the intensity and dynamics of emotional experiences (variability and instability) are particularly important. Emotional variability reflects the wide range of positive and negative emotions over successive time points (Van der Giessen et al., 2015); emotional instability is generally defined as showing frequent and intense emotional swings over time without correspondingly severe environmental events (Trull et al., 2008); finally, emotional intensity refers to the extent of emotional responses (Goto & Schaefer, 2020).

In the last decades, retrospective methods used for emotional assessments in people with mental disorders have been progressively replaced by the real‐time Experience Sampling Method (ESM) (Hektner et al., 2007). Experience Sampling Method aims to systematically obtain self‐report data on participants' everyday lives at many points in time in real‐world settings (Csikszentmihalyi & Larson, 1987; Hektner et al., 2007). Such rich data allows for inferring the dynamics of emotions in terms of an individual's emotional intensity as well as emotional variability and instability (Links et al., 2007). Experience Sampling Method holds the potential to serve as a valuable tool for mental health professionals, enabling them to gain a deeper understanding of their patients' emotional dynamics and providing insights into the impact of medication on emotional states. This, in turn, empowers professionals to offer personalized interventions that target individual emotional responses. By uncovering specific daily patterns and exploring associations with contextual factors, interventions can be focused on implementing coping strategies during specific times of the day. Additionally, by identifying specific emotional patterns or changes preceding relapse, clinicians can establish early warning systems or preventive strategies to intervene and mitigate the risk of relapse. Research also indicates that individuals with SSD often experience therapeutic benefits through monitoring their experiences and behaviors (Hanssen et al., 2020). By understanding their own emotional triggers and fluctuations, these individuals can develop a better comprehension of their condition and actively engage in their treatment process. Despite the increasing application of ESM in psychiatric research settings, to date, few studies have been performed to assess the emotions of people with SSD using this method. Indeed, the majority of the studies using ESM on this population have focused on the evaluation of the intensity of the emotional experience: these investigations have found that people with SSD experience more negative emotions (Cho et al., 2017; Sanchez et al., 2014) in their daily lives than healthy controls. By contrast, results about the positive emotions of these individuals are mixed: some studies found lower positive emotions among people with SSD than among healthy controls (Cho et al., 2017), while others found equivalent levels of positive emotions and enjoyment of the activity in people with SSD and healthy controls (Sanchez et al., 2014). In one recent study (Jones et al., 2021), participants with schizophrenia (*n* = 102) were compared to those with bipolar disorder (*n* = 71) on their experience of daily emotions (happy, sad, relaxed, and anxious) over up to 90 assessments over a 30‐day ESM sampling period. In this study, 18% of the participants with schizophrenia reported that they never underwent a single experience of sadness over the sampling period, while the other participants reported both positive and negative emotions that were consistent with those seen in participants with bipolar disorder. All participants were living in the community, thus the results cannot address the issues associated with more severe illness features requiring supervised residential care.

Therefore, in order to overcome the existing limitations of the literature on emotions in individuals with SSD, the objectives of this study were: (1) to evaluate between and within‐person differences between residential patients with SSD, outpatients with SSD, and healthy controls in both negative and positive emotional intensity, variability and instability in daily hours and weekdays, and investigate their clinical correlates; and (2) to assess the strength of the association between psychiatric severity/levels of functioning and emotional variability/instability in people with SSD. We hypothesized that people with SSD would report more negative emotional intensity, variability and instability than healthy controls, and they would also display significant associations between more impaired emotional processes and greater psychiatric severity and lower levels of functioning.

## 2 METHODS

### 2.1 Study setting

This multisite project (DiAPAson project ‐ Daily Activities, Physical Activities and Interpersonal relationships) (de Girolamo et al., 2020; Martinelli et al., 2022; Martinelli et al., 2023; Mayeli et al., 2023; Oliva et al., 2022; Zarbo et al., 2022; Zarbo, Rota, et al., 2023; Zarbo, Zamparini, Killaspy, et al, 2023; Zarbo, Zamparini, Nielssen, et al., 2023; Zarbo, Stolarski, et al., 2023) included 20 Departments of Mental Health (DMHs) and 98 Residential Facilities (RF) located in different Italian regions. Departments of Mental Health recruited both outpatient and residential patients, and RFs only residential patients. RFs had a mean number of 12.8 (SD = 5.7) residents, and each recruited a mean of 3.5 (SD = 2.6) patients, approximately 27% of patients in each RF. Since there were logistic and financial limitations, this ecological ESM study was conducted in a subsample of sites involved in the overall project: these include 7 DMHs, one clinical research center (IRCCS) and two RFs. With regard to RFs, recent, official data provided by the Italian Ministry of Health show that there are in Italy 1983 RFs hosting 27,813 people with mental disorders, with a rate of 5.7 people per 10,000 inhabitants. Half of the residents of Italian RFs are people with SSD (Ministero della Salute, 2022). RFs account for about 40% of the total Department of Mental Health costs, despite involving only 3.4% of all people in treatment at public mental health services. Although RFs are regulated by Italian national guidelines, they are somewhat heterogeneous in their approach with differing aims, rules, size, staffing, length of stay, environmental features, and target population, as shown in a large nationwide project on RFs conducted some years ago (de Girolamo et al., 2002, 2005).

From a methodological point of view, the choice to divide the two groups based on treatment setting (e.g., outpatients who live independently or patients who live in RFs with staff cover 24/24 h) was justified on the basis of the highly different environments across these two different settings: we hypothesize that this may have a direct impact on the participants' emotional patterns. From a clinical point of view, the identification of different emotional patterns among people living in different settings may facilitate the development of personalized treatment plans that allow for a wider range of scores in symptoms and function.

### 2.2 Study participants and procedures

We included individuals with a DSM‐5 diagnosis of SSD (Association & American Psychiatric Association, 2013). All SSD participants were 20–55 years old, able to speak and write in Italian, and in treatment at RFs or as outpatients at DMH. We excluded people with SSD who were unable to provide informed consent or who manifested severe cognitive deficits (i.e., a Mini‐Mental State Examination [MMSE] corrected score lower than 24), had a recent (of the last 6 months) diagnosis of substance use disorder according to DSM‐5 criteria (Association & American Psychiatric Association, 2013), had a history of clinically significant head injury, or cerebrovascular/neurological disease.

To reduce selection bias, outpatients (who were community‐dwelling people with SSD) were seen consecutively at the DMH until the achievement of the desired target sample for each site. Similarly, in the RFs, facility chiefs prepared an alphabetical list of patients with SSD present on an index day: based on this list, patients were consecutively invited to participate in the study until the required target sample was achieved. Healthy controls were recruited by public advertisement and snowball sampling procedures, and they were matched by gender and age group (i.e., 20–24, 25–29, 30–34, 35–39, 40–44, 45–49, and 50–55) with the clinical sample who completed the ESM ecological study. Healthy controls were not screened for mental health conditions.

Participants were provided with detailed information about the study and had the opportunity to ask questions. Some of the assessment tools were completed by the treating clinician, while Research Assistants (RA) helped the patients complete self‐reported questionnaires. All measures were completed across samples using the same methodology and standardized clinical measures were used to collect clinical data to minimize methodological biases. The ESM monitoring was preceded by a briefing session in which RAs gave instructions about the procedures and how to effectively perform them. The monitoring was followed by a debriefing section in which the same RA collected information on study acceptability and feasibility. During the debriefing session, outpatients and healthy controls received € 25,00 for travel expense reimbursement. All participants provided written informed consent and local Ethical Committees approved the study.

### 2.3 Clinical assessment

In this paper, we focus on the staff‐rated measures of disorder severity, negative symptoms, and levels of functioning. The 24‐item Brief Psychiatric Rating Scale (BPRS) (Morosini & Casacchia, 1995) was used to assess the presence and severity of psychopathology; BPRS items were rated on a seven‐point scale ranging from 1 (not present) to 7 (extremely severe). Negative symptoms were assessed with the Brief Negative Symptom Scale (BNSS) (Mucci et al., 2015; Strauss et al., 2012), a 13‐item instrument designed for the evaluation of blunted affect, alogia, asociality, anhedonia, and avolition (item scores ranged from 0, not present to 6, severe deficit). The 43‐item Specific Levels of Functioning Scale (SLOF) (Montemagni et al., 2015) was used to assess different aspects of daily functioning. The SLOF is a multidimensional behavioral assessment tool comprising six subscales: physical functioning, personal care skills, interpersonal relationships, social acceptability, activities of community living, and work skills. The SLOF items were rated on a five‐point scale ranging from 1 to 5, with higher ratings indicating higher functioning. These ratings were generated with information obtained from interviews with patients and clinicians.

### 2.4 Assessment of daily emotions with Experience Sampling Method

Daily emotions were prospectively assessed with a brief questionnaire on a smartphone‐based application for ESM, specifically developed for the project. The mobile application included three sections: current activities, social contacts, and emotions. The first section asked “*What are you doing right now?”* and the participants could choose between 15 activities. The second section asked “*Who are you with right now?*” with two options: “Alone” or “With other people”. The third section displayed seven emotional states in a random order (i.e., happy, sad, tired, relaxed, nervous, calm, and full of energy) and each subject had to rate that emotion at that moment on a scale of 0–100 (0 not at all ‐ 100 a lot). Positive emotions were computed as the mean of the following items: happy, relaxed, quiet, and full of energy. Negative emotions were computed as the mean of the following ESM items: sad, tired, and nervous. Notifications occurred 8 times a day, from 8 a.m. to 12 p.m., for 7 consecutive days. Positive and negative emotion scores were computed as the mean of the items in each category.

Notifications were semi‐randomized (i.e., randomly sent within eight scheduled time slots), and a reminder was sent after 15 min in case of no response to the initial prompt. Participants had a maximum of 30 min to answer.

### 2.5 Statistical analysis

Since incomplete data sets might bias statistical models used for analysis, participants who responded to less than 30% of the notifications (17/56) were excluded from the analyses. We only considered data from answered surveys (no imputation). Descriptive statistics include frequencies for categorical variables, and mean (and standard deviation [sd]), and median (and interquartile range) for continuous variables. Differences between groups were tested with chi‐square tests and multivariate analysis ANOVA (or non‐parametric tests, after assessing the distributions of continuous variables for normality with the Kolmogorov‐Smirnov test). The Bonferroni post‐hoc correction was applied for ANOVA results. Correlations between clinical variables (BPRS, BNSS, and SLOF) and emotion ratings were calculated with Spearman correlation coefficients.

Variability was considered between‐person, daily within‐person and within‐one‐week within‐person, calculating the mean Sum of Squared Differences using the standard definition of variance (the average of the squared differences from the mean). For the three different types of variance, the formulas are:

1. $Between-person\;variance=\frac{\sum_{i}{\left(X_{i}-\bar{X}\right)}^{2}}{n}$
  Where $\bar{X}$ = average value of the group (Residential patients, Outpatients and Healthy Controls).
***X***_***i***_ = average value of the *i*th subject.
***n*** = number of people in the group.
2. $Daily\;within-person\;variance=\frac{\sum_{i}\left(\frac{\sum_{j}{\left(X_{ij}-\bar{X_{i}}\right)}^{2}}{n}\right)}{m}$
  Where $\bar{X_{i}}$ = average value of the *i*th subject.
***X***_***ij***_ = average value of the *i*th subject in the *j*th time‐slot (1 = *08–10*; 2 = *10–12*…).
***n*** = number of time slots registered from the *i*th subject.
***m*** = number of people in the group.
3. $Weekly\;within-person\;variance=\frac{\sum_{i}\left(\frac{\sum_{j}{\left(X_{ij}-\bar{X_{i}}\right)}^{2}}{n}\right)}{m}$
  Where $\bar{X_{i}}$ = average value of the *i*th subject.
$X_{ij}$ = average value of the *i*th subject in the *j*th day (1 = *Monday*, 2 = *Tuesday*…).
***n*** = number of days registered from the *i*th subject.
***m*** = number of people in the group.

To establish emotional instability, successive squared differences between assessments were calculated. These were calculated only on assessments within the same days. Subsequent squared difference values were then square root transformed so that they reflected the size of the absolute change or successive difference in emotional levels between measurement occasions. Sociodemographic and clinical features of participants (e.g., illness duration, age, gender, group, BNSS, BPRS, and SLOF ratings), treatments (e.g., number of anti‐psychotics [APs], mood stabilizers, and antidepressants taken [No‐APs]) and average intensity of ESM emotional ratings (positive and negative emotions; ranging from 0 to 100) were entered in generalized linear models and selected through backward elimination to explain negative and positive variability and instability. Differences in diurnal and within‐one‐week trends were investigated: time of day and day of the week (time) were treated as continuous variables in these time‐trend models, first unadjusted and then adjusting estimates for potential confounders linked to emotions and daily/weekly routine (i.e., age and sex). Maximum likelihood procedures were used to account for missing observations in participants who met the minimum criteria for adherence. For confirmatory purposes we tested the correlation between Positive and Negative Emotion scores, to verify that they were inversely related.

All analyses were performed using SAS, *R*, and SPSS, considering a maximum I type error of 5% (I type error‐inflated results were managed with Bonferroni correction for multiple tests).

## 3 RESULTS

From October 2020 to October 2021, 673 eligible people with a diagnosis of SSD and aged 20–55 years (340 residentials, 333 outpatients) and 115 healthy controls (matched for age and sex with the clinical sample) were recruited. Among the 673 patients with SSD initially selected, 17 (2.5%) were excluded for severe cognitive impairment (i.e., MMSE <24), 36 patients (26 outpatients and 10 residentials, 5.3%) dropped out of the study, and 128 (19.1%) participated to the ESM study. Of them, 25 were excluded for not having reached the minimum compliance threshold of 30% of answered notifications. Therefore, the final sample included 103 participants with a diagnosis of SSD (57 residential patients, 46 outpatients) and 112 healthy controls (two healthy controls have not reached the minimum compliance threshold of 30%). Figure S1 shows the process of participant recruitment.

### 3.1 Sociodemographic and clinical characteristics of participants

The sociodemographic and clinical characteristics of participants are shown in Table 1. Most participants were males (63.1%). There was a significant difference in marital status, education, and employment between the three groups (*p* < 0.001).

**TABLE 1:** Sociodemographic and clinical features of the sample.

Variables	[1]	[2]	[3]	*p* value	Post‐hoc comparison
	Residential patients	Outpatients	Healthy controls		
	*N* = 57 (26.5%)	*N* = 46 (21.4%)	*N* = 112 (52.1%)		
Sex, *n* (%)					
Male	40 (70.2%)	27 (58.7%)	68 (60.7%)	0.393	
Age (mean, SD)	43.4 (9.9)	38.1 (10.4)	41.8 (10.0)	**0.028**	[2]<[1]
Median (IQR)	46.0 (15.0)	37.0 (17.0)	44.5 (17.0)		
Marital status, *n* (%)					
Single	46 (80.7%)	38 (82.6%)	27 (24.1%)	**<0.001** ^a^	
Married or cohabiting	5 (8.8%)	4 (8.7%)	78 (69.6%)		
Divorced or widowed	6 (10.5%)	4 (8.7%)	7 (6.3%)		
Education (mean, SD)	11.6 (3.6)	12.5 (2.4)	16.6 (4.9)	**<0.001** ^a^	[1]/[2]<[3]
Median (IQR)	11.0 (5.0)	13.0 (2.0)	17.0 (7.0)		
Working status, *n* (%)					
Working	8 (14.0%)	23 (50.0%)	103 (92.0%)	**<0.001** ^a^	
Studying	3 (5.3%)	6 (13.0%)	8 (7.1%)		
Not working/studying	46 (80.7%)	17 (37.0%)	1 (0.9%)		
Illness duration (mean, SD)	19.8 (10.5)	15.3 (9.0)	NA	**0.040**	‐‐
Median (IQR)	20.0 (15.0)	13.0 (16.0)	NA		
Lifetime duration of psychiatric hospitalizations, *n* (%)					
<1 year	10 (17.5%)	43 (93.5%)	NA	**<0.001** ^a^	
1–5 years	21 (36.8%)	1 (2.2%)	NA		
>5 years	26 (45.6%)	2 (4.3%)	NA		
APs drugs (mean, SD)	1.7 (0.8)	1.5 (0.8)	NA	0.118	‐‐
Median (IQR)	2.0 (1.0)	1.0 (1.0)	NA		
No‐APs drugs (mean, SD)	1.8 (1.3)	0.9 (0.8)	NA	**<0.001** ^a^	‐‐
Median (IQR)	2.0 (1.0)	1.0 (1.0)	NA		
BPRS (mean, SD)	48.4 (13.2)	40.8 (9.6)	NA	**0.003**	‐‐
Median (IQR)	47.0 (19.0)	40.0 (16.0)	NA		
BNSS^a^ (mean, SD)	24.1 (15.0)	17.0 (13.2)	NA	**0.016**	‐‐
Median (IQR)	23.0 (22.0)	14.0 (22.0)	NA		
SLOF^a^ (mean, SD)	175.1 (21.3)	188.5 (32.2)	NA	**<0.001** ^a^	‐‐
Median (IQR)	174.0 (29.0)	195.0 (23.0)	NA		
Positive emotions (mean, SD)	62.3 (20.2)	60.0 (17.1)	60.1 (11.9)	0.776	
Median (IQR)	56.6 (28.8)	57.7 (25.5)	57.7 (16.6)		
Negative emotions (mean, sd)	30.3 (15.9)	25.4 (14.1)	23.2 (10.4)	**0.012**	[3]<[1]
Median (IQR)	32.4 (20.7)	25.0 (16.5)	23.1 (16.2)		
Patients excluded (compliance ≤30%)	15/72	10/56	1/113	‐‐	‐‐

*Note*: Bold values highlight the *p* value <0.05.

Abbreviation: NA, Not Applicable.

^a^ Still significant after Bonferroni correction.

The two SSD groups differed for illness duration (*p* = 0.040), lifetime duration of inpatient psychiatric treatment (*p* < 0.001), number of No‐APs drugs (*p* < 0.001), BPRS ratings (*p* = 0.003), BNSS ratings (*p* = 0.016), and SLOF ratings (*p* < 0.001).

### 3.2 Emotional intensity

**FIGURE 1:**
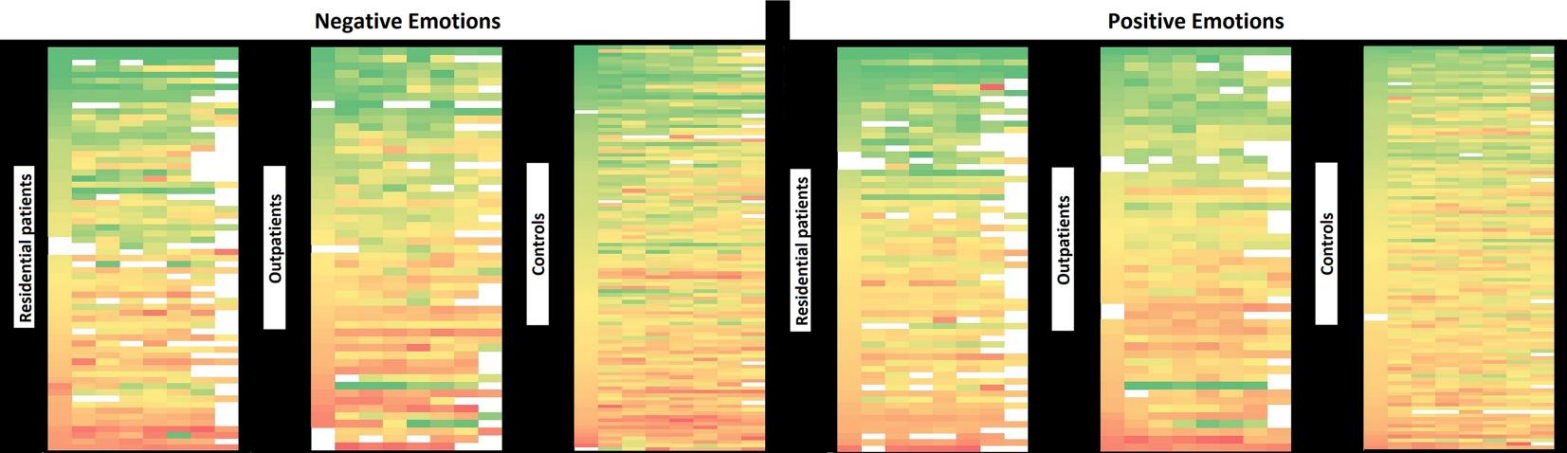
Color‐coded of Positive and Negative Emotions' ratings of patients with schizophrenia spectrum disorders (SSD) and healthy controls over the 7‐Day monitoring. Each row represents a subject, and each square represents an hourly slot (starting from left: 8–10, 10–12…). The colors range from dark green (corresponding to Positive Emotions between 80 and 100 and Negative Emotions between 0 and 20) to dark red (corresponding to Positive Emotions between 0 and 20 and Negative Emotions between 80 and 100).

**FIGURE 2:**
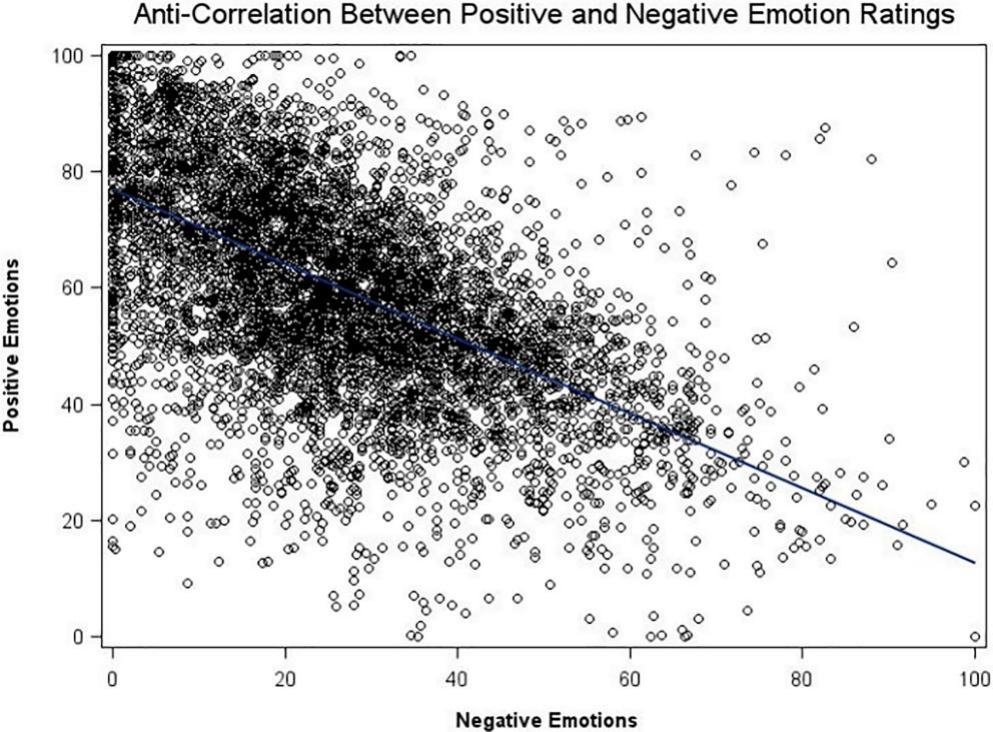
Anti‐correlation between Positive and Negative Emotion ratings. *r* = −0.578, *p* < 0.001. Each data point represents a single evaluation, with a total of 8578 filled prompts out of 13,496 hypothetically available prompts, resulting in a response rate of 63.6%. The evaluations were collected from 241 individuals who received 8 daily prompts over a period of 7 days; this number also includes those subjects who were subsequently excluded from the final analyses for not meeting the criterion of at least 30% of replies to daily prompts. As anticipated, there were minimal instances where participants simultaneously reported high levels of both Positive and Negative Emotions, as indicated by the scarcity of data points in the lower left and upper right regions of the graph.

With regard to positive emotional intensity, we found no significant differences between the three groups (residential patients: 62.3, SD = 20.2; outpatients: 60.0, SD = 17.1; healthy controls: 60.1, SD = 11.9; *p* 0.776). Moreover, both residential patients (30.3, SD = 15.9) and outpatients (25.4, SD = 14.1) experienced a similar intensity of negative emotions. Also, outpatients did not significantly differ from healthy controls (23.2, SD = 10.4) in terms of negative emotional intensity. However, residential patients experienced a greater intensity of negative emotions than healthy controls (*p* = 0.012) (Table 1, Figure 1). The strongly negative correlation between positive and negative emotional intensity confirms the reliability of the data (Figure 2).

### 3.3 Emotional variability and instability

**FIGURE 3:**
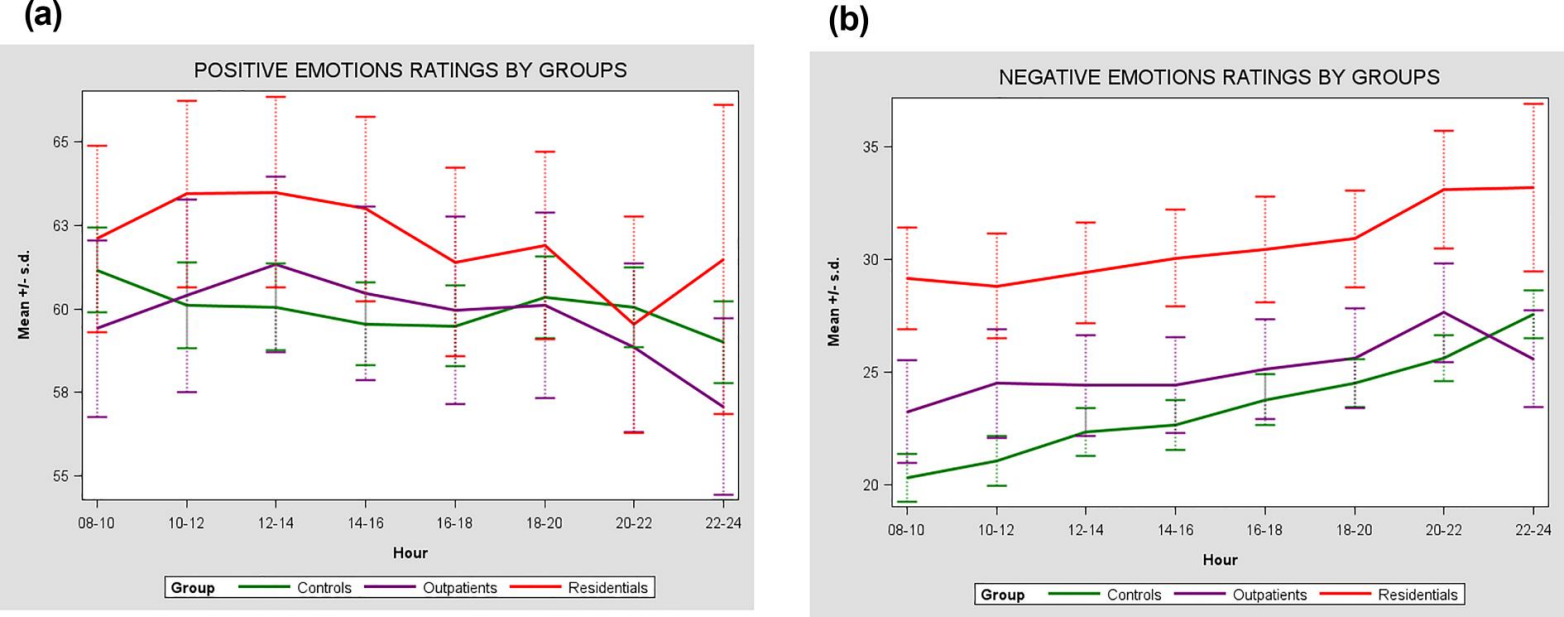
(a) Individual differences in emotions mean ratings between residential patients with schizophrenia spectrum disorders (SSD), outpatients with SSD and healthy controls during the daily hours. (b) Individual differences in emotions mean ratings between residential patients with SSD, outpatients with SSD and healthy controls during the daily hours.

**FIGURE 4:**
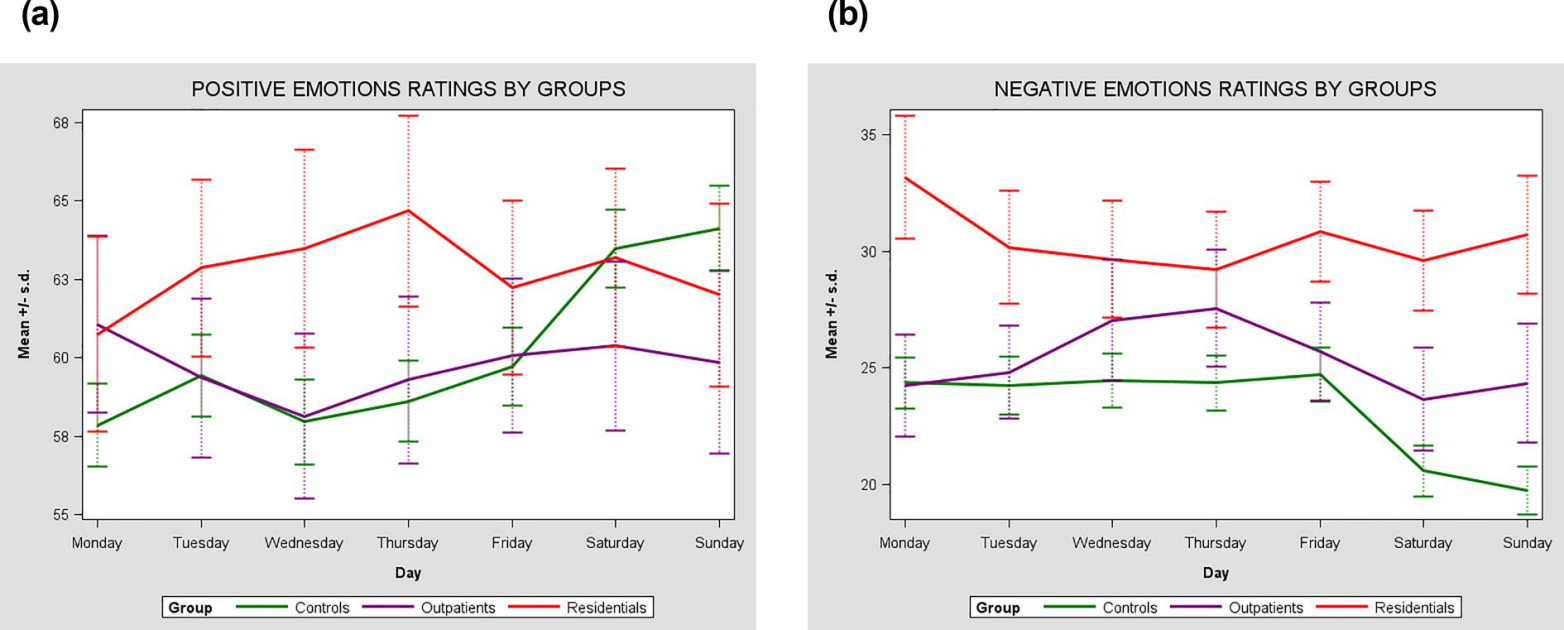
(a) Individual differences in emotions mean ratings between residential patients with schizophrenia spectrum disorders (SSD), outpatients with SSD, and healthy controls during the week. (b) Individual differences in emotions mean ratings between residential patients with SSD, outpatients with SSD, and healthy controls during the week.

Between‐person emotional variability (i.e., the emotional variability within each group) showed statistically significant differences in both positive and negative emotions between the 3 groups (Table 2, Figures 3 and 4). The highest variability in positive emotions emerged in residential patients (*σ* ^2^ = 407.5 [95% CI 290.5–613.3]), compared to outpatients (*σ* ^2^ = 292.3 [95% CI 201.1–469.8]) and controls (*σ* ^2^ = 141.4 [95% 110.5–187.5] *p* < 0.001). Moreover, higher variability in negative emotions' ratings was found in both residential patients (*σ* ^2^ = 252.7 [95% CI 180.1–380.3]) and outpatients (*σ* ^2^ = 198.2 [95% CI 136.4–314.5]) compared to controls (*σ* ^2^ = 107.2 [95% CI 83.7–142.0]; *p* < 0.001) (Table 3).

**TABLE 2:** Between‐person variability, within‐person (daily and within‐one‐week) variability, and instability for Experience Sampling Method (ESM) emotions' ratings.

	Residential patients	Outpatients	Healthy controls	*p* value	Post‐hoc comparison
	*N* = 57	*N* = 46	*N* = 112		
Between‐person variability [95% CI]					
Positive emotions	407.5 [290.5; 613.3]	292.3 [201.1; 469.8]	141.4 [110.5; 187.5]	**<0.001** ^a^	Controls < Outpatients < Residentials
Negative emotions	252.7 [180.1; 380.3]	198.2 [136.4; 314.5]	107.2 [83.7; 142.0]	**<0.001** ^a^	Controls < Outpatients/Residentials
Within‐person (daily) variability [95% CI]					
Positive emotions	52.6 [13.1; 92.2]	40.7 [23.3; 58.0]	36.4 [30.5; 42.4]	0.198	
Negative emotions	50.5 [36.1; 65.0]	50.8 [30.8; 70.8]	31.9 [26.9; 37.0]	**0.013**	Controls < Outpatients/Residentials
Within‐person (within‐one‐week) variability [95% CI]					
Positive emotions	70.1 [42.3; 97.9]	40.7 [30.5; 50.9]	60.6 [50.3; 70.9]	**<0.001** ^a^	Outpatients < Residentials
Negative emotions	69.7 [44.9; 94.5]	48.0 [29.5; 66.5]	48.5 [40.3; 56.6]	**0.004**	Outpatients/Controls < Residentials
Instability [95% CI]					
Positive emotions	9.17 [7.64; 10.70]	9.24 [7.96; 10.51]	9.82 [9.12; 10.53]	0.250	
Negative emotions	10.53 [8.93; 12.13]	9.06 [7.82; 10.30]	9.05 [8.38; 9.73]	0.149	

*Note*: Bold values highlight the *p* value <0.05.

^a^ Still significant after Bonferroni correction.

**TABLE 3:** Correlation Matrix between psychiatric severity/psychosocial functioning and emotional instability and variability (daily and weekly) in patients with schizophrenia spectrum disorders (SSD).

	BPRS	BNSS	SLOF
Residential patients and outpatients *Rho (p)*			
Intraday variability of positive emotions	−0.02 (0.853)	−0.04 (0.698)	−0.16 (0.111)
Intraday variability of negative emotions	−0.02 (0.853)	0.04 (0.721)	−0.20 (0.046)
Intraweek variability of positive emotions	0.19 (0.051)	0.04 (0.674)	−0.25 (0.010)
Intraweek variability of negative emotions	0.20 (0.038)	0.21 (0.035)	−0.32 (0.001^a^)
Instability of positive emotions	0.13 (0.175)	−0.01 (0.930)	−0.14 (0.148)
Instability of negative emotions	0.17 (0.086)	0.22 (0.028)	−0.31 (0.002)
Residential patients *Rho (p)*			
Intraday variability of positive emotions	0.02 (0.908)	−0.14 (0.314)	−0.10 (0.465)
Intraday variability of negative emotions	−0.07 (0.958)	−0.04 (0.754)	−0.15 (0.272)
Intraweek variability of positive emotions	0.13 (0.353)	−0.09 (0.491)	−0.20 (0.142)
Intraweek variability of negative emotions	0.22 (0.101)	0.13 (0.332)	−0.25 (0.063)
Instability of positive emotions	0.12 (0.367)	−0.02 (0.902)	−0.16 (0.228)
Instability of negative emotions	0.12 (0.363)	0.20 (0.131)	−0.26 (0.053)
Outpatients *Rho (p)*			
Intraday variability of positive emotions	0.04 (0.780)	0.14 (0.336)	−0.22 (0.139)
Intraday variability of negative emotions	−0.02 (0.898)	0.13 (0.377)	−0.20 (0.179)
Intraweek variability of positive emotions	0.35 (0.018)	0.25 (0.096)	−0.34 (0.023)
Intraweek variability of negative emotions	0.18 (0.242)	0.31 (0.036)	−0.41 (0.006)
Instability of positive emotions	0.22 (0.149)	0.02 (0.915)	−0.14 (0.356)
Instability of negative emotions	0.13 (0.377)	0.13 (0.393)	−0.18 (0.243)

^a^ Still significant after Bonferroni correction.

For within‐person daytime emotional variability, no significant differences emerged between the three groups for positive emotions (*p* = 0.198) although there were significant differences between the three groups in the daily variability of negative emotions. Both residential patients (*σ* ^2^ = 50.5 [95% CI 36.1–65.0]) and outpatients (*σ* ^2^ = 50.8 [95% CI = 30.8–70.8]) had greater daily variability than controls (*σ* ^2^ = 31.9 [95% = 26.9–37.0] *p* = 0.013).

The within‐person emotional variability across the days of the week was significantly different in the two patient groups (*p* < 0.001): residential patients had greater day to day variability in positive emotions (*σ* ^2^ = 70.1 [95% CI 42.3–97.9]) than outpatients (*σ* ^2^ = 40.7 [95% CI 30.5–50.9]). Residential patients also showed greater day to day variability in negative emotions (*σ* ^2^ = 69.7 [95% CI 44.9–94.5]) compared to both outpatients (*σ* ^2^ = 48.0 [95% CI 29.5–66.5]) and controls (*σ* ^2^ = 48.5 [ 95% CI 40.3; 56.6] *p* = 0.004).

Finally, we found no significant differences in terms of emotional instability (i.e., the difference between successive ratings) between the three groups with regard to both positive (*p* 0.250) and negative (*p* 0.149) emotions.

### 3.4 Correlation between psychiatric severity/levels of functioning and emotion instability and variability

The variability of negative emotions from day to day within the week was significantly and positively associated with the psychiatric symptoms measured with the BPRS (*r* = 0.20; *p* = 0.038) and negative symptoms measured with the BNSS (*r* = 0.21; *p* = 0.035), and negatively associated with everyday functioning indexed by SLOF ratings (*r* = −0.32; *p* = 0.001). Specific Levels of Functioning Scale ratings were also negatively correlated to within‐day variability of negative emotions (*r* = −0.20; *p* = 0.046), intraweek variability of positive emotions (*r* = −0.25; *p* = 0.010) and instability of negative emotions (*r* = −0.31; *p* = 0.002). Brief Negative Symptom Scale ratings were positively associated with the instability of negative emotions (*r* 0.22; *p* = 0.028).

### 3.5 Predictors of emotional variability and instability

We found a significant positive effect of the number of APs drugs on both intraday (*β* = 27.5 [95% CI 2.4–52.6]; *p* = 0.032) and intraweek (*β* = 24.3 [95% CI 6.5–42.2]; *p* = 0.008) variability of positive emotions among individuals with SSD. Moreover, a higher number of mood stabilizers predicted higher both intraday (*β* = 106.4 [95% CI 69.0–143.8]; *p* < 0.001) and intraweek (*β* = 66.8 [95% CI 40.0–93.6]; *p* < 0.001) variability of positive emotions, as well as higher intraweek variability of negative emotions (*β* = 33.8 [95% CI 4.0–63.5]; *p* 0.026). Specific Levels of Functioning Scale ratings negatively predicted the instability of negative emotions, (*β* = −0.1 [95% CI −0.11–0.02]; *p* = 0.006), while BNSS ratings significantly predicted the intraday variability of negative emotions (*β* = 0.9 [95% CI 0.1–1.7]; *p* = 0.025). Positive coefficients indicate an increase in variability, suggesting less coherence of emotions over time. On the other hand, negative coefficients imply a decrease in variability, indicating better emotional constancy (Table 4).

**TABLE 4:** Predictors of emotions instability and within‐person variability (daily and weekly).

(Ref: Controls, male)	*β*	95% CI	*p*
Intraday variability of positive emotions			
Illness duration	−1.7	−3.7; 0.2	0.085
AP	27.5	2.4; 52.6	**0.032**
Mood stabilizers	106.4	69.0; 143.8	**<0.001** ^a^
Intraday variability of negative emotions			
BNSS	0.9	0.1; 1.7	**0.025**
Intraweek variability of positive emotions			
AP	24.3	6.5; 42.2	**0.008**
Mood stabilizers	66.8	40.0; 93.6	**<0.001** ^a^
Intraweek variability of negative emotions			
Negative emotions	1.1	0.6; 2.1	**0.039**
SLOF	−0.7	−1.5; 0.1	0.067
Mood stabilizers	33.8	4.0; 63.5	**0.026**
Instability of positive emotions			
Negative emotions	0.1	0.01; 0.14	**0.034**
Instability of negative emotions			
Negative emotions	0.1	0.05; 0.18	**0.001** ^a^
SLOF	−0.1	−0.11; −0.02	**0.006**
Female	1.8	−0.2; 3.7	0.080

*Note*: Ranges: Illness duration (1–40); AP (0–4); Mood stabilizers (0–3); BNSS (0–55); Negative Emotions (0–64); SLOF (125–215). Bold values highlight the *p* value <0.05.

^a^ Still significant after Bonferroni correction.

### 3.6 Effects of hours and days on emotional intensity

The intensity of positive and negative emotions as reported by residential patients and outpatients did not show any time‐related trend traceable using linear or quadratic models (Table 5).

**TABLE 5:** Estimates of linear and quadratic time of day effects (unadjusted and adjusted for gender and age) on Experience Sampling Method (ESM) emotions ratings.

Unadjusted	Residential patients			Outpatients			Healthy controls		
	Coeff.	95% CI	*p*	Coeff.	95% CI	*p*	Coeff.	95% CI	*p*
Positive emotions									
Time (Hour)	−0.38	−1.33; 0.58	0.438	−0.30	−1.14; 0.55	0.490	−0.17	−0.55; 0.21	0.377
Time^2^(Hour)	−0.05	−0.16; 0.06	0.400	−0.04	−0.13; 0.05	0.386	−0.02	−0.06; 0.03	0.427
Time (Day)	0.11	−0.97; 1.20	0.838	0.01	−0.99; 1.00	0.987	1.03	0.54; 1.51	**<0.001** ^a^
Time^2^ (Day)	0.00	−0.13; 0.13	0.977	0.01	−0.11; 0.13	0.895	0.13	0.08; 0.19	**<0.001** ^a^
Negative emotions									
Time (Hour)	0.62	−0.15; 1.39	0.112	0.45	−0.25; 1.15	0.204	0.97	0.64; 1.30	**<0.001** ^a^
Time^2^(Hour)	0.07	−0.01; 0.16	0.097	0.05	−0.03; 0.13	0.221	0.11	0.07; 0.14	**<0.001** ^a^
Time (Day)	−0.26	−1.15; 0.62	0.560	−0.11	−0.97; 0.75	0.803	−0.75	−1.17; −0.33	**0.001** ^a^
Time^2^ (Day)	−0.02	−0.13; 0.09	0.712	−0.03	−0.13; 0.08	0.612	−0.10	−0.15; −0.05	**<0.001** ^a^

Adjusted	Residential patients			Outpatients			Healthy controls		
	Coeff.	95% CI	*p*	Coeff.	95% CI	*p*	Coeff.	95% CI	*p*
Positive emotions									
Time (Hour)	−0.41	−1.36; 0.55	0.399	−0.30	−1.14; 0.55	0.492	−0.17	−0.54; 0.20	0.360
Time^2^(Hour)	−0.05	−0.16; 0.06	0.357	−0.04	−0.14; 0.05	0.388	−0.02	−0.06; 0.02	0.413
Time (Day)	0.10	−0.98; 1.19	0.850	0.01	−0.99; 1.00	0.996	1.03	0.55; 1.50	**<0.001** ^a^
Time^2^ (Day)	0.01	−0.13; 0.13	0.990	0.01	−0.12; 0.13	0.904	0.13	0.08; 0.19	**<0.001** ^a^
Negative emotions									
Time (Hour)	0.66	−0.09; 1.42	0.083	0.44	−0.26; 1.14	0.215	0.97	0.64; 1.30	**<0.001** ^a^
Time^2^(Hour)	0.08	−0.01; 0.17	0.067	0.05	−0.03; 0.12	0.232	0.11	0.07; 0.14	**<0.001** ^a^
Time (Day)	−0.25	−1.12; 0.62	0.570	−0.12	−0.98; 0.74	0.787	−0.75	−1.17; −0.33	**0.001** ^a^
Time^2^ (Day)	−0.02	−0.13; 0.09	0.728	−0.03	−0.13; 0.08	0.596	−0.10	−0.15; −0.05	**<0.001** ^a^

*Note*: Bold values highlight the *p* value <0.05.

^a^ Still significant after Bonferroni correction.

However, healthy controls reported both a linear (*β* 1.03 [95% CI 0.55–1.50] *p* < 0.001) and quadratic (*β* ^2^ 0.13 [95% CI 0.08–0.19] *p* < 0.001) trend of an increase in positive emotions from Monday to Sunday, whereas the intensity of their negative emotions increased going from morning to evening (linear time: *β* 0.97 [95% CI 0.64:1.30]; *p* < 0.001; quadratic time: *β* ^2^ 0.11 [95% CI 0.07–0.14]; *p* < 0.001) and decreased over the week (Linear time: *β* −0.75 [95% CI −1.17–0.33]; *p* = 0.001; quadratic time: *β* ^2^ −0.10 [95% CI −0.15–0.05]; *p* < 0.001).

## 4 DISCUSSION

This study found that people with SSD, particularly when living in residential settings, tend to show higher negative emotional intensity, within‐group variability of both positive and negative emotions, and variability during the day and the week of negative emotions than healthy controls. We found that intraday and intraweek emotional variability in people with SSD did not follow a linear or quadratic trend and were significantly related to psychiatric symptoms, levels of functioning, and current pharmacological treatment.

### 4.1 Do individuals with schizophrenia spectrum disorders show a higher intensity of negative emotions compared to healthy controls?

Our study shows that individuals with SSD are more likely to report higher intensity of negative emotions compared to the general population, confirming the results of previous research (Myin‐Germeys et al., 2000; Sanchez et al., 2014). For a long time, the main focus of research on people with SSD has been on cognitive symptoms and positive psychotic symptoms (i.e., hallucinations and delusions), with less attention devoted to emotional factors, despite the established finding of emotional impairment being a central feature of the overall clinical picture (Fakra et al., 2015). Emotional impairment and its dysregulation in these people with SSD may be due to a defect in the recognition of emotions and their expression (Fakra et al., 2008, 2009, 2015).

A recent article by Jones et al. (2021) suggests that the reduced ability to subjectively evaluate emotional experience among individuals with SSD may be considered a core feature of negative symptoms. Several studies have also shown how, unlike healthy controls, people with SSD can experience emotions that are not consistent with environmental stimuli (Larsen et al., 2001), conveying negative affect even in the presence of positive and neutral stimuli (Cohen & Minor, 2010; Park et al., 2008; Strauss & Herbener, 2011; Trémeau et al., 2009; Ursu et al., 2011).

### 4.2 Do individuals with schizophrenia spectrum disorders experience greater emotional variability than healthy controls?

Our study highlights that people with SSD have greater between‐person variability in the experience of emotions (i.e., people with SSD are more different from each other) when compared to healthy controls. Moreover, people with SSD seem to show higher emotional variability than healthy controls in different timeframes, during the day and during the week. These results are consistent with a study comparing individuals with schizophrenia and healthy controls showing that the former are more cyclothymic (Fichtner et al., 1989). This observation could be attributed to the possibility that individuals with schizophrenia often encounter a decline in social, occupational, or recreational engagements. This reduction may stem from factors such as diminished motivation or obstacles of a financial or social nature. However, these results are inconsistent with those of Dalkner et al. (Dalkner et al. (2023), who reported no significant effects of day or time of day in modulating the variability of negative emotions among outpatients with SSD. This inconsistency may be due to the fact that the above‐mentioned study included only outpatients, while our study found greater variability of negative emotions (especially across the days) among residential patients. Therefore, residential patients, who have more severe impairment from a psychosocial standpoint, should be considered an important subgroup that needs specific attention.

In addition, we found that the variability in people with SSD does not follow a linear or quadratic trend, but their emotional experience is more variable and chaotic compared to healthy controls, who displayed a linear or quadratic pattern of emotional variability over time. Indeed, the controls showed greater regularity of emotional experience within the hours of the day and the days of the week. Participants with schizophrenia have previously been described to report reduced positive emotional states when leaving their residence and an increase in these emotions when they returned, compared to healthy controls who were happier when away from home (Parrish et al., 2020). Research had shown that regardless of gender, job position, and salary, people consistently feel better (mentally and physically) over the weekend. This finding seems likely related to the typical week structure of healthy people: weekdays are dedicated to working activities, while the weekend tends to be associated with leisure time spent in rewarding or relaxing activities and social interactions. We can hypothesize that, similarly to the weekly trend, the linearity in the emotional expression of healthy people during the day is due to a greater daily structure, with an evening increase in subjective negative emotions probably due to the fact that during the first hours of the day individuals are more relaxed and rested. This linear variability in healthy people can also be mediated by well‐being indicators such as autonomy and relationships, which are generally lacking in individuals with SSD, especially in residential patients who have very limited autonomy. The different structures of daily life may partly explain the chaotic pattern of emotional variability found during the week in participants with SSD.

### 4.3 Are individuals with more severe schizophrenia spectrum disorders also more unstable and variable in their emotional expression?

We found that participants receiving residential treatment also exhibited the greatest variability and emotional instability (especially negative emotions). Moreover, the data demonstrate a correlation between a higher usage of antipsychotics and mood stabilizers and increased emotional variability throughout the day and week. It is essential to note that this correlation may be confounded by indication, as people with more severe SSD often receive higher medication doses. Therefore, it is crucial to emphasize that the observed association does not imply causality. Moreover, while medication adherence for residential patients is ensured, this is not true for outpatients, who may be non‐adherent. More medication prescribed does not mean more medication taken, so inferences regarding the number and dose of medications from prescription data in the absence of adherence indicators such as serum levels or observed medication adherence. Many studies have shown that antipsychotic medications have minimal effects on neurocognitive and social‐cognitive variables (Kee et al., 1998; Keefe, 2007; Littrell et al., 2004) and also on negative symptoms and emotional functioning. Further, momentary environmental factors, such as current location (home vs. away) and social context (with someone vs. alone), may in part explain the variation of emotional states in this population (Harvey et al., 2021) and need further investigation.

### 4.4 Limitations

This study has some limitations. First, the time of data acquisition: the groups were assessed from October 2020 to October 2021 during the COVID‐19 pandemic, which led to containment measures, including prolonged lockdown, which may have influenced the emotional state of all participants. Indeed, it is possible that the clinical status and the emotional severity, variability, and instability of participants may have been affected by the pandemic, exacerbating between‐group differences. Another limitation concerns the exclusion of older individuals from the survey, focusing on the young‐adult population. Therefore, it is possible that older participants, especially if suffering from SSD, may show different daily emotional patterns and emotional predictors due to different physical, psychiatric and social statuses (e.g., higher social isolation, increased number of physical co‐morbidities, more prolonged illness status).

Based on previous knowledge and pragmatic choices, we were able to assess only a limited number of emotions, and some negative emotions (e.g., anger) were uncovered by our study. Additional investigations in the future may shed light on other negative emotions.

## 5 CONCLUSIONS

The use of an ecological, real‐time assessment method (ESM) has made it possible to carry out a fine‐grained investigation of the emotional intensity, variability and instability in individuals with SSD during a week, overcoming the limitations related to the traditional summary measures of retrospective recollection of emotional states. This study suggests two key methodological insights for future research: (1) the significance of evaluating emotional patterns in individuals with severe mental disorders, encompassing not only SSD but also other types such as bipolar disorders, borderline personality disorders, depression, etc., utilizing dynamic, real‐time assessment methods like ESM, as opposed to static, retrospective approaches; and (2) the necessity of establishing connections between changes in emotional patterns over time and meaningful life events, which encompass not only stressful occurrences but also encompass daily events or positive experiences.

## AUTHOR CONTRIBUTIONS

**Cristina Zarbo**: Conceptualization; data curation; formal analysis; investigation; methodology; project administration; supervision; roles/writing – original draft; writing – review & editing. **Manuel Zamparini**: Conceptualization; data curation; formal analysis; methodology; supervision; writing – original draft; writing – review & editing. **Alessandra Patrono**: Conceptualization; methodology; roles/writing – original draft; writing – review & editing. **Cosima Calini**: Conceptualization; methodology; roles/writing – original draft; writing – review & editing. **Philip D. Harvey**: Writing – original draft; writing – review & editing; supervision. **Letizia Casiraghi**: Investigation; data curation; writing – review & editing. **Massimo Clerici**: Writing – original draft; writing – review & editing; supervision. **Matteo Rocchetti**: Conceptualization; funding acquisition; methodology; supervision; writing – review & editing. **Fabrizio Starace**: Conceptualization; funding acquisition; methodology; supervision; writing – review & editing. **Giovanni de Girolamo**: Conceptualization; funding acquisition; methodology; project administration; supervision; writing – original draft; writing – review & editing.

## CONFLICT OF INTEREST STATEMENT

The authors declare that they have no competing interest.

## ETHICS STATEMENT

The study has been approved by the Ethical Committees (ECcs) of the three main participating centers (EC of IRCCS Istituto Centro San Giovanni di Dio Fatebenefratelli, July 31, 2019; no. 211/2019; EC of Area Vasta Emilia Nord, 25/September 2019; no. 0025975/19), and (EC of ASST of Pavia, September 02, 2019, no. 20190075685) and by the ECs of all participating sites.

## Supporting information

Supplementary Information S1

## Data Availability

Dataset referring to this manuscript is published with restricted access on Zenodo platform and accessible at this link: https://doi.org/10.5281/zenodo.7050149.

## References

[mpr1992-bib-0001] American Psychiatric Association . (2013). Diagnostic and statistical manual of mental disorders, DSM‐5 (5th edition). 10.1176/appi.books.9780890425596

[mpr1992-bib-0002] Berenbaum, H. , & Oltmanns, T. F. (1992). Emotional experience and expression in schizophrenia and depression. Journal of Abnormal Psychology, 101(1), 37–44. 10.1037/0021-843x.101.1.37 1537971 PMC4370316

[mpr1992-bib-0003] Cho, H. , Gonzalez, R. , Lavaysse, L. M. , Pence, S. , Fulford, D. , & Gard, D. E. (2017). Do people with schizophrenia experience more negative emotion and less positive emotion in their daily lives? A meta‐analysis of experience sampling studies. Schizophrenia Research, 183, 49–55. 10.1016/j.schres.2016.11.016 27881233

[mpr1992-bib-0004] Cohen, A. S. , & Minor, K. S. (2010). Emotional experience in patients with schizophrenia revisited: meta‐analysis of laboratory studies. Schizophrenia Bulletin, 36(1), 143–150. 10.1093/schbul/sbn061 18562345 PMC2800132

[mpr1992-bib-0005] Correll, C. U. , & Schooler, N. R. (2020). Negative symptoms in schizophrenia: A review and clinical guide for recognition, assessment, and treatment. Neuropsychiatric Disease and Treatment, 16, 519–534. 10.2147/ndt.s225643 32110026 PMC7041437

[mpr1992-bib-0006] Csikszentmihalyi, M. , & Larson, R. (1987). Validity and reliability of the experience‐sampling method. The Journal of Nervous and Mental Disease, 175(9), 526–536. 10.1097/00005053-198709000-00004 3655778

[mpr1992-bib-0007] Dalkner, N. , Moore, R. C. , Depp, C. A. , Ackerman, R. A. , Pinkham, A. E. , & Harvey, P. D. (2023). Negative mood states as a correlate of cognitive performance and self‐assessment of cognitive performance in bipolar disorder versus schizophrenia. Schizophrenia Research, 252, 1–9. 10.1016/j.schres.2022.12.034 36608492 PMC9974828

[mpr1992-bib-0008] de Girolamo, G. , Picardi, A. , Micciolo, R. , Falloon, I. , Fioritti, A. , & Morosini, P. , & for the Italian PROGRES study group . (2002). Residential care in Italy: A national survey of non‐hospital facilities. British Journal of Psychiatry, 181(3), 220–225. 10.1192/bjp.181.3.220 12204926

[mpr1992-bib-0009] de Girolamo, G. , Rocchetti, M. , Benzi, I. M. A. , Agosta, S. , Casiraghi, L. , Ferrari, C. , De Franceschi, N. , Macis, A. , Pogliaghi, S. , & Starace, F. (2020). DAily time use, physical activity, quality of care and interpersonal relationships in patients with schizophrenia spectrum disorders (DiAPASon): An Italian multicentre study. BMC Psychiatry, 20(1), 287. 10.1186/s12888-020-02588-y 32513140 PMC7278132

[mpr1992-bib-0010] de Girolamo, G. , Picardi, G. A. , Santone, G. , Falloon, I. , Morosini, P. , Fioritti, A. , & Micciolo, R. , & for the PROGRES Group . (2005). The severely mentally ill in residential facilities: A national survey in Italy. Psychological Medicine, 34, 1–11. 10.1017/s0033291704003502 15841877

[mpr1992-bib-0011] Fakra, E. , Belzeaux, R. , Azorin, J.‐M. , & Adida, M. (2015). [Negative symptoms, emotion and cognition in schizophrenia]. L’Encephale, 41(6 Suppl 1), 6S18–6S21. 10.1016/s0013-7006(16)30005-7 26776386

[mpr1992-bib-0012] Fakra, E. , Khalfa, S. , Da Fonseca, D. , Besnier, N. , Delaveau, P. , Azorin, J. M. , & Blin, O. (2008). Effect of risperidone versus haloperidol on emotional responding in schizophrenic patients. Psychopharmacology, 200(2), 261–272. 10.1007/s00213-008-1203-y 18575849

[mpr1992-bib-0013] Fakra, E. , Salgado‐Pineda, P. , Besnier, N. , Azorin, J.‐M. , & Blin, O. (2009). Risperidone versus haloperidol for facial affect recognition in schizophrenia: Findings from a randomised study. World Journal of Biological Psychiatry: The Official Journal of the World Federation of Societies of Biological Psychiatry, 10(4 Pt 3), 719–728. 10.1080/15622970701432536 17853271

[mpr1992-bib-0014] Fichtner, C. G. , Grossman, L. S. , Harrow, M. , Goldberg, J. F. , & Klein, D. N. (1989). Cyclothymic mood swings in the course of affective disorders and schizophrenia. American Journal of Psychiatry, 146(9), 1149–1154. 2764171 10.1176/ajp.146.9.1149

[mpr1992-bib-0015] Goto, N. , & Schaefer, A. (2020). Emotional intensity. In Encyclopedia of personality and individual differences (pp. 1311–1319). 10.1007/978-3-319-24612-3_509

[mpr1992-bib-0016] Hanssen, E. , Balvert, S. , Oorschot, M. , Borkelmans, K. , van Os, J. , Delespaul, P. , & Fett, A.‐K. (2020). An ecological momentary intervention incorporating personalised feedback to improve symptoms and social functioning in schizophrenia spectrum disorders. Psychiatry Research, 284, 112695. 10.1016/j.psychres.2019.112695 31831201

[mpr1992-bib-0017] Harvey, P. D. , Miller, M. L. , Moore, R. C. , Depp, C. A. , Parrish, E. M. , & Pinkham, A. E. (2021). Capturing clinical symptoms with ecological momentary assessment: Convergence of momentary reports of psychotic and mood symptoms with diagnoses and standard clinical assessments. Innovations in Clinical Neuroscience, 18(1–3), 24–30. PMC8195558 34150360

[mpr1992-bib-0018] Hektner, J. M. , Schmidt, J. A. , & Csikszentmihalyi, M. (2007). Experience sampling method: Measuring the quality of everyday life. SAGE.

[mpr1992-bib-0019] Jones, S. E. , Moore, R. C. , Depp, C. A. , Ackerman, R. A. , Pinkham, A. E. , & Harvey, P. D. (2021). Daily Ecological Momentary Assessments of happy and sad moods in people with schizophrenia and bipolar disorders: What do participants who are never sad think about their activities and abilities? Schizophrenia Research. Cognition, 26, 100202. 10.1016/j.scog.2021.100202 34189061 PMC8219985

[mpr1992-bib-0020] Kahn, R. S. , Sommer, I. E. , Murray, R. M. , Meyer‐Lindenberg, A. , Weinberger, D. R. , Cannon, T. D. , O’Donovan, M. , Correll, C. U. , Kane, J. M. , van Os, J. , & Insel, T. R. (2015). Schizophrenia. Nature Reviews Disease Primers, 1, 15067. 10.1038/nrdp.2015.67 27189524

[mpr1992-bib-0021] Kee, K. S. , Kern, R. S. , Marshall, B. D., Jr. , & Green, M. F. (1998). Risperidone versus haloperidol for perception of emotion in treatment‐resistant schizophrenia: Preliminary findings. Schizophrenia Research, 31(2–3), 159–165. 10.1016/s0920-9964(98)00026-7 9689720

[mpr1992-bib-0022] Keefe, R. S. E. (2007). Neurocognitive effects of antipsychotic medications in patients with chronic schizophrenia in the CATIE trial. Archives of General Psychiatry, 64(6), 633. 10.1001/archpsyc.64.6.633 17548746

[mpr1992-bib-0023] Kring, A. M. , Kerr, S. L. , Smith, D. A. , & Neale, J. M. (1993). Flat affect in schizophrenia does not reflect diminished subjective experience of emotion. Journal of Abnormal Psychology, 102(4), 507–517. 10.1037/0021-843x.102.4.507 8282918

[mpr1992-bib-0024] Kring, A. M. , & Neale, J. M. (1996). Do schizophrenic patients show a disjunctive relationship among expressive, experiential, and psychophysiological components of emotion? Journal of Abnormal Psychology, 105(2), 249–257. 10.1037/0021-843x.105.2.249 8723006

[mpr1992-bib-0025] Larsen, J. T. , McGraw, A. P. , & Cacioppo, J. T. (2001). Can people feel happy and sad at the same time? Journal of Personality and Social Psychology, 81(4), 684–696. 10.1037/0022-3514.81.4.684 11642354

[mpr1992-bib-0026] Links, P. S. , Eynan, R. , Heisel, M. J. , Barr, A. , Korzekwa, M. , McMain, S. , & Ball, J. S. (2007). Affective instability and suicidal ideation and behavior in patients with borderline personality disorder. Journal of Personality Disorders, 21(1), 72–86. 10.1521/pedi.2007.21.1.72 17373891

[mpr1992-bib-0027] Littrell, K. H. , Petty, R. G. , Hilligoss, N. M. , Kirshner, C. D. , & Johnson, C. G. (2004). Improvement in social cognition in patients with schizophrenia associated with treatment with olanzapine. Schizophrenia Research, 66(2–3), 201–202. 10.1016/s0920-9964(03)00185-3 15061258

[mpr1992-bib-0062] Martinelli, A. , D’Addazio, M. , Zamparini, M. , Thornicroft, G. , Torino, G. , Zarbo, C. , Rocchetti, M. , Starace, F. , Casiraghi, L. , Ruggeri, M. , & de Girolamo, G. (2023). Needs for care of residents with schizophrenia spectrum disorders and association with daily activities and mood monitored with experience sampling method: The DIAPASON study. Epidemiology and Psychiatric Sciences, 32. 10.1017/s2045796023000124 PMC10130736 37039434

[mpr1992-bib-0065] Martinelli, A. , Killaspy, H. , Zarbo, C. , Agosta, S. , Casiraghi, L. , Zamparini, M. , Starace, F. , Rocchetti, M. , de Girolamo, G. , Ruggeri, M. , Barlati, S. , Boero, M. E. , Cerveri, G. , Clerici, M. , D’Anna, G. , De Novellis, A. , Di Michele, V. , Di Prisco, P. , Durbano, F. , … Tura, G. (2022). Quality of residential facilities in Italy: satisfaction and quality of life of residents with schizophrenia spectrum disorders. BMC Psychiatry, 22(1). 10.1186/s12888-022-04344-w PMC9672559 36397009

[mpr1992-bib-0056] Mayeli, A. , LaGoy, A. D. , Smagula, S. F. , Wilson, J. D. , Zarbo, C. , Rocchetti, M. , Starace, F. , Zamparini, M. , Casiraghi, L. , Calza, S. , Rota, M. , D’Agostino, A. , de Girolamo, G., & Ferrarelli, F. (2023). Shared and distinct abnormalities in sleep‐wake patterns and their relationship with the negative symptoms of Schizophrenia Spectrum Disorder patients. Molecular Psychiatry. 10.1038/s41380-023-02050-x 37055512

[mpr1992-bib-0028] Ministero della Salute . (2022). Rapporto salute mentale. Analisi dei dati del Sistema Informativo per la Salute Mentale (SISM). Anno 2021. Ministero della Salute.

[mpr1992-bib-0029] Montemagni, C. , Rocca, P. , Mucci, A. , Galderisi, S. , & Maj, M. (2015). Italian version of the “specific level of functioning”. Journal of Psychopathology and Behavioral Assessment, 21(3), 287–296.

[mpr1992-bib-0057] Morosini, P. L. , & Casacchia, M. (1995). Traduzione italiana della brief psychiatric rating scale, versione 4.0 ampliata (BPRS 4.0). Rivista di riabilitazione Psichiatrica e Psicosociale, 3, 199–228.

[mpr1992-bib-0031] Mucci, A. , Galderisi, S. , Merlotti, E. , Rossi, A. , Rocca, P. , Bucci, P. , Piegari, G. , Chieffi, M. , Vignapiano, A. , & Maj, M. (2015). The Brief Negative Symptom Scale (BNSS): Independent validation in a large sample of Italian patients with schizophrenia. European Psychiatry, 30(5), 641–647. 10.1016/j.eurpsy.2015.01.014 25758156

[mpr1992-bib-0032] Myin‐Germeys, I. , Delespaul, P. A. , & de Vries, M. W. (2000). Schizophrenia patients are more emotionally active than is assumed based on their behavior. Schizophrenia Bulletin, 26(4), 847–854. 10.1093/oxfordjournals.schbul.a033499 11087017

[mpr1992-bib-0033] Myin‐Germeys, I. , Nicolson, N. A. , & Delespaul, P. A. (2001). The context of delusional experiences in the daily life of patients with schizophrenia. Psychological Medicine, 31(3), 489–498. 10.1017/s0033291701003646 11305857

[mpr1992-bib-0063] Oliva, V. , Fanelli, G. , Zamparini, M. , Zarbo, C. , Rocchetti, M. , Casiraghi, L. , Starace, F. , Martinelli, A. , Serretti, A. , & de Girolamo, G. (2022). Patterns of antipsychotic prescription and accelerometer‐based physical activity levels in people with schizophrenia spectrum disorders: A multicenter, prospective study. International Clinical Psychopharmacology, 38(1), 28–39. 10.1097/yic.0000000000000433 36165505 PMC9722380

[mpr1992-bib-0034] Park, I. H. , Park, H.‐J. , Chun, J.‐W. , Kim, E. Y. , & Kim, J.‐J. (2008). Dysfunctional modulation of emotional interference in the medial prefrontal cortex in patients with schizophrenia. Neuroscience Letters, 440(2), 119–124. 10.1016/j.neulet.2008.05.094 18562102

[mpr1992-bib-0035] Parrish, E. M. , Depp, C. A. , Moore, R. C. , Harvey, P. D. , Mikhael, T. , Holden, J. , Swendsen, J. , & Granholm, E. (2020). Emotional determinants of life‐space through GPS and ecological momentary assessment in schizophrenia: What gets people out of the house? Schizophrenia Research, 224, 67–73. 10.1016/j.schres.2020.10.002 33289659 PMC13085614

[mpr1992-bib-0036] Sanchez, A. H. , Lavaysse, L. M. , Starr, J. N. , & Gard, D. E. (2014). Daily life evidence of environment‐incongruent emotion in schizophrenia. Psychiatry Research, 220(1–2), 89–95. 10.1016/j.psychres.2014.07.041 25124684 PMC4252781

[mpr1992-bib-0037] Strauss, G. P. , & Herbener, E. S. (2011). Patterns of emotional experience in schizophrenia: Differences in emotional response to visual stimuli are associated with clinical presentation and functional outcome. Schizophrenia Research, 128(1–3), 117–123. 10.1016/j.schres.2011.01.010 21330110 PMC3085645

[mpr1992-bib-0038] Strauss, G. P. , Keller, W. R. , Buchanan, R. W. , Gold, J. M. , Fischer, B. A. , McMahon, R. P. , Catalano, L. T. , Culbreth, A. J. , Carpenter, W. T. , & Kirkpatrick, B. (2012). Next‐generation negative symptom assessment for clinical trials: Validation of the brief negative symptom scale. Schizophrenia Research, 142(1–3), 88–92. 10.1016/j.schres.2012.10.012 23127378 PMC3502630

[mpr1992-bib-0039] Trémeau, F. , Antonius, D. , Cacioppo, J. T. , Ziwich, R. , Jalbrzikowski, M. , Saccente, E. , Silipo, G. , Butler, P. , & Javitt, D. (2009). In support of bleuler: Objective evidence for increased affective ambivalence in schizophrenia based upon evocative testing. Schizophrenia Research, 107(2–3), 223–231. 10.1016/j.schres.2008.09.020 18947981

[mpr1992-bib-0040] Trull, T. J. , Solhan, M. B. , Tragesser, S. L. , Jahng, S. , Wood, P. K. , Piasecki, T. M. , & Watson, D. (2008). Affective instability: Measuring a core feature of borderline personality disorder with ecological momentary assessment. Journal of Abnormal Psychology, 117(3), 647–661. 10.1037/a0012532 18729616

[mpr1992-bib-0041] Upthegrove, R. , Marwaha, S. , & Birchwood, M. (2016). Depression and schizophrenia: Cause, consequence or trans‐diagnostic issue? In Schizophrenia bulletin. 10.1093/schbul/sbw097.sbw097. PMC5605248 27421793

[mpr1992-bib-0042] Ursu, S. , Kring, A. M. , Gard, M. G. , Minzenberg, M. J. , Yoon, J. H. , Ragland, J. D. , Solomon, M. , & Carter, C. S. (2011). Prefrontal cortical deficits and impaired cognition‐emotion interactions in schizophrenia. American Journal of Psychiatry, 168(3), 276–285. 10.1176/appi.ajp.2010.09081215 21205806 PMC4019338

[mpr1992-bib-0043] Van der Giessen, D. , Hollenstein, T. , Hale, W. W., 3rd , Koot, H. M. , Meeus, W. , & Branje, S. (2015). Emotional variability in mother‐adolescent conflict interactions and internalizing problems of mothers and adolescents: Dyadic and individual processes. Journal of Abnormal Child Psychology, 43(2), 339–353. 10.1007/s10802-014-9910-9 25070359

[mpr1992-bib-0044] Winship, I. R. , Dursun, S. M. , Baker, G. B. , Balista, P. A. , Kandratavicius, L. , Maia‐de‐Oliveira, J. P. , Hallak, J. , & Howland, J. G. (2019). An overview of animal models related to schizophrenia. Canadian Journal of Psychiatry. Revue Canadienne de Psychiatrie, 64(1), 5–17. 10.1177/0706743718773728 29742910 PMC6364139

[mpr1992-bib-0064] Zarbo, C. , Agosta, S. , Casiraghi, L. , De Novellis, A. , Leuci, E. , Paulillo, G. , Rocchetti, M. , Starace, F. , Zamparini, M. , & de Girolamo, G. (2022). Assessing adherence to and usability of Experience Sampling Method (ESM) and actigraph in patients with Schizophrenia Spectrum Disorder: A mixed‐method study. Psychiatry Research, 314, 114675. 10.1016/j.psychres.2022.114675 35751998

[mpr1992-bib-0058] Zarbo, C. , Rota, M. , Calza, S. , Crouter, S. E. , Ekelund, U. , Barlati, S. , Bussi, R. , Clerici, M. , Placenti, R. , Paulillo, G. , Pogliaghi, S. , Rocchetti, M. , Ruggeri, M. , Starace, F. , Zanolini, S. , Zamparini, M. , & de Girolamo, G. (2023). Ecological monitoring of physical activity, emotions and daily life activities in schizophrenia: The DiAPAson study. BMJ Mental Health, 26(1), e300836. 10.1136/bmjment-2023-300836 PMC11146405 37666578

[mpr1992-bib-0061] Zarbo, C. , Stolarski, M. , Zamparini, M. , Damiani, S. , Casiraghi, L. , Rocchetti, M. , Starace, F. , & de Girolamo, G. (2023). Time perspective affects daily time use and daily functioning in individuals with Schizophrenia Spectrum Disorders: Results from the multicentric DiAPAson study. Journal of Psychiatric Research, 160, 93–100. 10.1016/j.jpsychires.2023.02.012 36796292

[mpr1992-bib-0059] Zarbo, C. , Zamparini, M. , Killaspy, H. , Baldini, V. , Patrono, A. , Malvezzi, M. , Casiraghi, L. , Rocchetti, M. , Starace, F. , & de Girolamo, G. (2023). Daily time use among individuals with schizophrenia spectrum disorders and unaffected controls: Results from the DiAPAson multicentric project. Psychiatric Rehabilitation Journal. 10.1037/prj0000576 37589695

[mpr1992-bib-0060] Zarbo, C. , Zamparini, M. , Nielssen, O. , Casiraghi, L. , Rocchetti, M. , Starace, F. , & de Girolamo, G. (2023). Comparing adherence to the experience sampling method among patients with schizophrenia spectrum disorder and unaffected individuals: Observational study from the multicentric DiAPAson project. Journal of Medical Internet Research, 25, e42093. 10.2196/42093 37463030 PMC10394602

