# Supplementary figures and images for: Ecological monitoring of emotional intensity, variability, and instability in individuals with schizophrenia spectrum disorders: Results of a multicentre study
Source: Int J Methods Psychiatr Res. 2023 Sep 20;33(1):e1992. doi: 10.1002/mpr.1992 (PMC10804261; doi:10.1002/mpr.1992)

## Figure 1S.

## Flowchart of sampling strategy


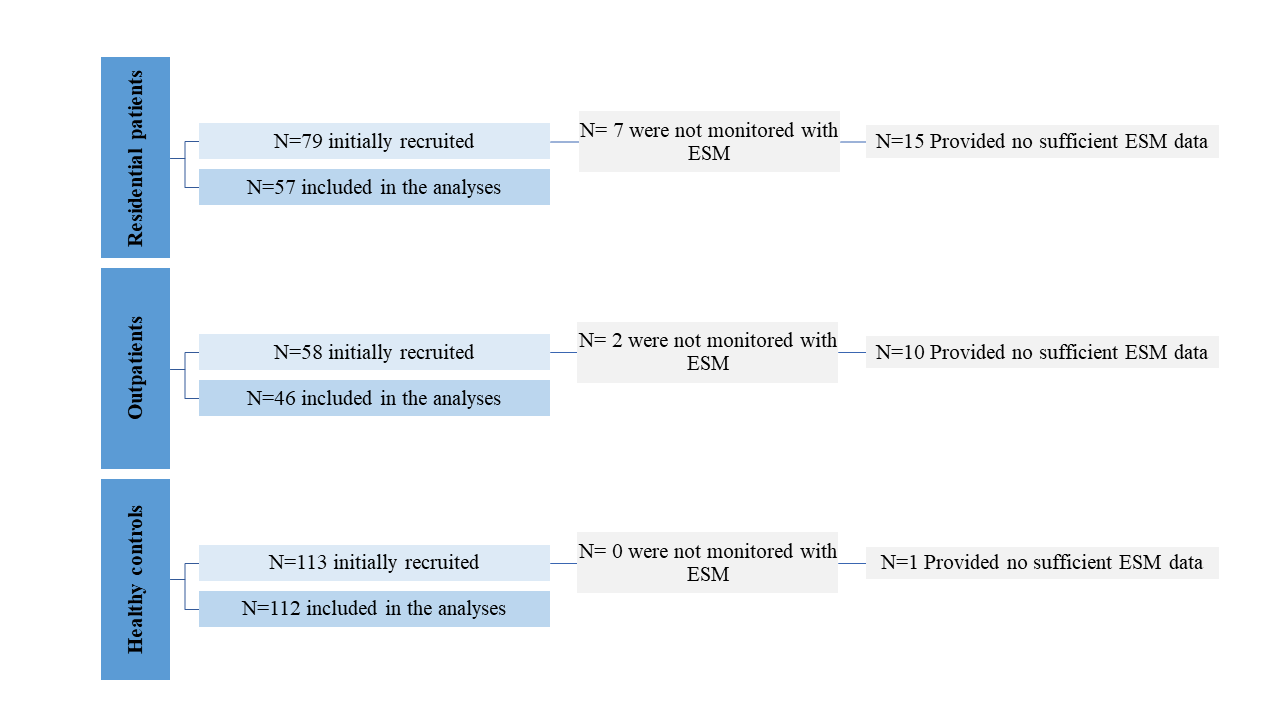

Supplement: Supplementary file 1 — Supplementary Information S1 [file MPR-33-e1992-s001.docx]
